# The effect of ice slurry ingestion on intermittent activity and a run to exhaustion in the heat

**DOI:** 10.1186/2046-7648-4-S1-A5

**Published:** 2015-09-14

**Authors:** Sarah Jackson, Nicola Gerrett

**Affiliations:** 1 Institute of Sport and Exercise Science, University of Worcester, UK

## Introduction

Major competitions are often staged in hot environments. However, the body's ability to dissipate heat is compromised during heat stress resulting in a concomitant rise in core temperature (T_c_), ultimately leading to impaired performance. Precooling methods such as ice vests and cold water immersion are employed before competition, to lower T_c _and increase heat storage before a critical T_c _is reached. However, there are numerous practical limitations meaning pre-existing methods are impractical for use in a field setting. Ice slurry ingestion has been investigated as a more practical precooling method and has enhanced endurance performance [1], however research has yet to determine its effects upon intermittent activity. Therefore the aim of this study was to determine the efficacy of ice slurry ingestion as a precooling method prior to intermittent activity and a run to exhaustion in the heat.

## Methods

In a counterbalanced order, ten female footballers (age 20 ± 1 years, height 162 ± 9.5 cm, weight 62.4 ± 8.5 kg, body fat 23.7 ± 2.6 %, body surface area 1.66 ± 0.15 m^2^, VO_2 _max 46.7 ± 4.6 mL.kg^-1^.min^-1^) either ingested 7.5 g.kg^−1 ^ice slurry (IS) or acted as a control (C). After ingestion a 45 minute intermittent shuttle test (walk 4 Km.h^-1 ^- 7 mins, jog 8 Km.h^-1 ^- 10 mins, cruise 10 Km.h^-1 ^- 5 mins, sprint 14 Km.h^-1 ^- _~_3 mins) was performed, followed by an alternating run to exhaustion (1 min at 65% VO_2 _max, 1 min at 95% until fatigue) in 33°C 59% rh. T_c_, skin temperature (T_sk_), heart rate (HR), rating of perceived exertion (RPE), thermal sensation and blood lactate (BLa) were measured during intermittent activity and T_c _and HR were recorded throughout the run to exhaustion. Gross sweat loss, heat storage and body temperature (T_b_) was calculated for the entire protocol. A student paired *t*-test was performed upon run time and sweat rate. A 2 × 2 repeated measures ANOVA was conducted upon T_c_, T_sk_, T_b_, HR, RPE, thermal sensation and BLa. Significance was set at P ≤ 0.05.

## Results

Time to exhaustion was significantly longer after ice slurry ingestion when compared to control (P = 0.0005). Prior to exercise ice slurry reduced T_c _by 0.3-1.0 °C (P = 0.034). T_c _remained significantly lower on IS compared with C during the first 30 minutes of intermittent activity (P = 0.05). Heat storage was significantly higher (P = 0.003), T_sk_, T_b _and RPE were significantly lower (P ≤ 0.05), and there was a trend for a reduction in thermal sensation throughout intermittent activity in the IS trial (P = 0.064). There were no significant differences between conditions in BLa (P = 0.743), HR (P = 0.113) and sweat rate (P = 0.278). At the end of the run to exhaustion T_c _was 0.3 °C higher on the IS trial (P = 0.004).

## Discussion

Ice slurry ingestion improved run time by 2 minutes 20 seconds (93 seconds), through reducing T_c _and promoting greater heat storage without any changes in blood lactate accumulation. Previous research indicates this could be due to numerous mechanisms including the reduction of inhibitory afferent feedback to the brain and stimulation of sensory receptors leading to improved perceptual responses.

## Conclusion

Ice slurry ingestion appears to be an effective precooling strategy for use in sports characterised by intermittent activity. Ice slurries are a practical alternative to existing methods and could be incorporated into pre-match hydration strategies. Future research should focus on determining the optimal volume and timing of ice slurry ingestion.
